# Performance Comparison of Different Approaches in Genotyping MHC-DRB: The Contrast between Single-Locus and Multi-Locus Species

**DOI:** 10.3390/ani12182452

**Published:** 2022-09-16

**Authors:** Ida Svetličić, Dean Konjević, Elena Bužan, Miljenko Bujanić, Luka Duniš, Sunčica Stipoljev, Jelena Martinčić, Mihaela Šurina, Ana Galov

**Affiliations:** 1 Department of Biology, Faculty of Science, University of Zagreb, Rooseveltov trg 6, 10000 Zagreb, Croatia; 2 Faculty of Veterinary Medicine, University of Zagreb, Heinzelova 55, 10000 Zagreb, Croatia; 3 Environmental Protection College, Trg mladosti 7, 3320 Velenje, Slovenia; 4 Faculty of Mathematics, Natural Sciences and Information Technologies, University of Primorska, Glagoljaška 8, 6000 Koper, Slovenia; 5 Faculty of Agriculture, University of Zagreb, Svetošimunska cesta 25, 10000 Zagreb, Croatia

**Keywords:** *Capreolus capreolus*, *Cervus elaphus*, Illumina, Ion Torrent, major histocompatibility complex, next-generation sequencing

## Abstract

**Simple Summary:**

Genes of the major histocompatibility complex (MHC) have been extensively used for estimation of genetic diversity in wild vertebrate populations on account of their exceptionally high polymorphism and key role in pathogen resistance. The complexity of the MHC region varies greatly, even between closely related species, and consequently influences the choice of genotyping strategy. Here, we compared and evaluated MHC genotyping in a single-locus species, the roe deer, and red deer, a species with multiple loci, by utilisation of molecular cloning and two high-throughput sequencing platforms (Illumina and Ion Torrent). For high-throughput data processing, we applied a web version of the Amplicon Sequencing Analysis Tools that analyses the first 5000 reads per sample as well as its locally installed script that analyses a total number of reads per sample, up to a maximum of 200,000. We observed genotype discrepancies only in red deer, with Illumina sequencing scoring the maximum number of detected alleles, regardless of the number of reads used for data analysis. This study facilitates the adoption of an optimal strategy for MHC genotyping in wild mammals that does not include complex bioinformatic analyses.

**Abstract:**

Major histocompatibility complex (MHC) genes are widely recognised as valuable markers for wildlife genetic studies given their extreme polymorphism and functional importance in fitness-related traits. Newly developed genotyping methods, which rely on the use of next-generation sequencing (NGS), are gradually replacing traditional cloning and Sanger sequencing methods in MHC genotyping studies. Allele calling in NGS methods remains challenging due to extreme polymorphism and locus multiplication in the MHC coupled with allele amplification bias and the generation of artificial sequences. In this study, we compared the performance of molecular cloning with Illumina and Ion Torrent NGS sequencing in MHC-DRB genotyping of single-locus species (roe deer) and species with multiple DRB loci (red deer) in an attempt to adopt a reliable and straightforward method that does not require complex bioinformatic analyses. Our results show that all methods work similarly well in roe deer, but we demonstrate non-consistency in results across methods in red deer. With Illumina sequencing, we detected a maximum number of alleles in 10 red deer individuals (42), while other methods were somewhat less accurate as they scored 69–81% of alleles detected with Illumina sequencing.

## 1. Introduction

Over the past few decades, major histocompatibility complex (MHC) genes have become a preferred marker for many wildlife population studies due to their functional importance in pathogen recognition and other fitness-related adaptations. The main feature of the MHC genes is their extreme genetic variability, indicated by a large number of divergent alleles in a population accompanied by an excess of heterozygosity and variation in loci number between and within species [1,2,3,4]. Given their complexity and extreme polymorphism, accurate genotyping of MHC loci in non-model species remains a difficult task, even with the advent of new technologies and approaches [4,5,6,7,8,9]. Previous research on roe deer MHC found evidence of only one DRB locus but has also acknowledged the frequent occurrence of insertion/deletion events in the detected alleles [10,11,12]. In red deer, the number of identified DRB loci differed across studied populations, ranging from one to four [13,14,15,16,17] and proved challenging for DRB genotyping with the traditional method.

The first methodological challenge in the assessment of individual MHC alleles is the design of target-specific primers [8], often hindered by the high sequence similarity between loci and the presence of duplications that lead to multi-locus allele amplification [5]. Primer design is followed by the choice of sequencing strategy, which can be divided into traditional and newly developed methods.

Traditional methods are commonly represented by cloning and the Sanger sequencing approach [18,19]. However, Sanger sequencing cannot provide sequencing information for a single DNA molecule, and therefore, in heterozygote individuals, allelic phases have to be resolved via cloning vector and individually sequenced. This approach is usually labour-intensive and costly, particularly when genotyping a large sample set of species with locus multiplication [20]. Furthermore, sequencing an insufficient number of clones can result in allelic dropout, particularly if the exact number of loci is unknown.

Newly developed genotyping methods rely on the use of NGS targeted sequencing to produce a vast number of sequences (termed “reads”) per individual, thus enabling the identification of all amplified gene variants [6,9,21]. NGS platforms differ in sequencing approaches and vary in achievable read lengths and depths, and technology-dependent error profiles. At present, Illumina platforms are the dominant technology for short-read sequencing, with a distinctive capability for paired-end sequencing and a maximum read length of 2 × 300 bp. Illumina employs the sequencing by synthesis approach, in which the incorporation of each nucleotide coupled with a reversible fluorescent terminator is detected by optical imaging [22]. In contrast, Ion Torrent sequencing by synthesis technology measures and records a change in pH caused by the release of hydrogen ions during nucleotide incorporation [23]. This sequencing technology offers a longer continuous read length in comparison with Illumina, reaching up to 400 bp [24]. NGS methods are considerably less time-consuming and more cost-efficient than traditional methods since they offer parallel sequencing and genotyping of large sample sets for a fraction of the price of cloning and Sanger sequencing [4,25]. A major challenge in these types of methods is the formation of chimeric sequences and other artefacts, which are present at a relatively high frequency in comparison with true alleles. Even though ambiguous sequences can be produced during standard PCR and cloning [3,21], they are more common in high throughput sequencing, making it more difficult to distinguish between true alleles and artefacts [6]. Consolidation of traditional and NGS methods can serve as a good strategy for preliminary MHC genotyping, enabling reliable and consistent future results [4].

The aim of this study was to compare traditional sequencing strategies with high-throughput methods in genotyping the DRB locus of MHC class II genes and contrast obtained results between two deer species with a different number of DRB copies—European roe deer, *Capreolus capreolus*, and red deer, *Cervus elaphus*. Specifically, we aimed to compare and evaluate cloning/Sanger sequencing with two NGS platforms (Illumina MySeq and IonTorrent S5 System) to reveal their potential in detecting true genotypes using the straightforward protocol for high-throughput data processing implemented in Amplicon Sequencing Analysis Tools (AmpliSAT) [26] in an effort to enable the adoption of a reliable and efficient method, which could be used in future genotyping projects, even by researchers with limited bioinformatics experience. Finally, because our initial analyses of red deer data yielded inconsistent results between methods, we further aimed to compare results obtained using the AmpliSAS web tool with those obtained using the locally installed AmpliSAS script.

## 2. Materials and Methods

Fourteen roe deer and ten red deer samples were used for this research. They were selected from a set of muscle and liver tissue samples that were collected as a part of a larger project on species adaptive diversity and host–parasite interactions from animals culled during regular management operations in Croatian hunting grounds and stored at approximately −20 °C in 96% ethanol. Ethical approval for this study was obtained from the Committee for Veterinary Ethics of the Veterinary Faculty University of Zagreb (Class: 640-01118-17/60, Ref. No.: 251-61-44-18-02). DNA extraction from 5–10 mg of each sample was performed using a Wizard Genomic DNA Purification Kit (Promega, Maidson, WI, USA). Initial analyses were comprised of polymerase chain reactions (PCR) used to specifically amplify a segment of exon 2 of the MHC-DRB gene. For this, we used the HotStarTaq DNA Polymerase kit (Qiagen, Hilden, Germany), with the PCR reactions prepared according to the manufacturer’s instructions. The primers we used were originally designed for cattle MHC, LA31 (5′-GATCCTCTCTCTGCAGCACATTTCCT-3′), and LA32 (5′-TTCGCGTCACCTCGCCGCTG-3′) [27], and were previously successfully used in roe deer [10,11,12] and red deer [15,17,28]. PCR was conducted in a total reaction volume of 40 μL, including 150–250 ng DNA, 0.2 μM of each primer, 2× HotStarTaq PCR buffer (including dNTPs, MgSO_4_, and Taq polymerase). Thermocycling comprised an initial denaturation step at 95 °C for 10 min, followed by 33 cycles of 1 min denaturation at 96 °C, 1 min of annealing at 58 °C, and 3 min of extension at 72 °C. A final extension step was performed at 72 °C for 15 min. Purification and Sanger sequencing of PCR products were performed by Macrogen Europe (Netherlands). Received sequences were inspected using BioEdit (Hall, 1999) [29] and SeqScape^®®^ (Applied Biosystems, Waltham, MA, USA) software. Upon initial analysis completion, the aforementioned heterozygote samples were selected for this research. Cloning of the selected samples was performed using the pGEM-T Vector System II (Promega, Maidson, WI, USA) and competent *Escherichia coli* JM 109 cells (Invitrogen, Carlsbad, CA, USA), following the manufacturer’s protocol. We isolated 30 recombinant clones per red deer and 15 recombinant clones per roe deer individual. Purified plasmids were sent for Sanger sequencing to the Macrogen Europe facility. The presence of two mosaic sequences was detected after cloning in one sample, and they were removed from further analysis. Illumina MiSeq paired-end PE250 sequencing was conducted at the Novogene facility (UK). PCRs were performed using LA31 and LA32 primers with specific barcodes. The construction of DNA libraries consisted of end repairing, followed by A-tailing, ligation of Illumina adapters, purification, and sequencing. Quality control consisted of a Nanodrop sample purity test, examination of DNA degradation and contamination through agarose gel electrophoresis, and Qubit 2.0 DNA quantification, conducted at each step of the procedure. Ion Torrent Amplicon sequencing was conducted using the Ion Torrent S5 system (Thermo Fisher Scientific, Waltham, MA, USA) following the methodology by Bužan et al. [12]. First, long PCR was carried out in triplicate, utilising the LA31 primer containing appropriate IonXpress barcodes and adapters, as well as the LA32 primer with the P1 adapter, which serves for the binding to ISP particles during the emulsion PCR. Next, PCR products belonging to the same sample were pooled, purified with AgencourtAMPure XP magnetic beads (Agencourt Bioscience Corporation, Beverly, MA, USA), and quantified with Qubit 3.0. Then, all amplicons were normalised, pooled, and purified again. Quality control and size of the library were verified with a 2100 Bioanalyzer Instrument (Agilent, Santa Clara, CA, USA) and normalised to 100 pM. Finally, the fragments were bound to ISP particles, amplified in the emulsion PCR, and sequenced on a 314 chip (Thermo Fisher Scientific, Waltham, MA, USA). Merging of the reads, quality filtering, and genotyping of individuals were performed using the AmpliSAT integrated web tools [26], available at http://evobiolab.biol.amu.edu.pl/amplisat/. The AmpliMERGE tool was used for merging the Illumina paired-end read files, while other tools were used for processing both Illumina and ion Torrent amplicon sequencing data. AmpliCLEAN was used for initial quality (Phred score >30) and size filtering. To preliminary inspect the data sets, the AmpliCHECK tool was utilised for the potential error annotations and assessment of the sequence lengths of putative alleles, which can serve as input data for the following genotyping performed by AmpliSAS. The genotyping algorithm consists of sequence demultiplexing, clustering, and filtering of the erroneous variants using user-defined parameters. Default AmpliSAS parameters were selected for each sequencing technology (Illumina: 1% substitution errors, 0.001% indel errors; Ion Torrent 0.5% substitution errors, 1% indel errors), with the minimum per amplicon frequency threshold of 1%. Since the web version of the AmpliSAS tool only utilises the first 5000 sample reads, the genotyping process was repeated with the same parameters using the AmpliSAS script installed locally to analyse all reads with a maximum of 200,000 reads per sample. This approach enabled the evaluation of both methods for accurate genotyping of red deer individuals. AmpliSAS analysis was not repeated for roe deer using locally installed scripts since cloning and sequencing on both platforms yielded identical results after initial genotyping with the web version. The efficiency of each method in detecting MHC-DRB alleles was tested through comparison with the combined genotype of each individual. A combined genotype consists of summed alleles obtained with the combination of all methods. The efficiency of each method was expressed as the proportion (P) of the combined genotype detected. Alleles obtained by each method were aligned and translated into amino acid sequences using BioEdit [29], and neither frameshifts nor stop codons were identified in any of the detected sequences.

## 3. Results

### 3.1. Roe Deer

The same DRB alleles were detected by all three methods (cloning/Sanger sequencing, Illumina, and Ion Torrent sequencing) in each of the 14 roe deer individuals, and no more than two alleles were found per individual (Table 1). Each allele in each individual was detected in at least one recombinant clone. 

After size and quality filtering, Illumina sequencing resulted in 1,202,786 reads and Ion Torrent resulted in 876,347 reads, while the average number of reads per sample after size and quality filtering was 85,913 for Illumina sequencing and 62,596 for Ion Torrent sequencing. The average proportion of reads assigned to alleles was very similar between Illumina and Ion Torrent (86.1% and 85.8%, respectively) (Supplementary file: Table S1). Individual read counts per detected allele ranged from 1580 to 2703 for Illumina and for Ion Torrent, ranged from 1008 to 3446. Allele frequencies within individuals were roughly in the expected ratio of 1:1 after Illumina sequencing, with the largest disproportion in sample L2 where the frequency ratio was 54.1%:43.1%. Allele frequency ratios were more diverse in Ion Torrent sequencing and the largest disproportion was in the sample L5, where the allele frequency ratio was 20.6%:64.5% (Supplementary file: Figure S1).

In total, eight alleles were detected, with lengths of either 246 or 249 bp (Supplementary file: Figure S2), seven of which were previously known. Allele Caca-DRB*0405 was found for the first time and was deposited in GenBank under the accession number ON204042. All detected alleles code for unique amino acid sequences.

### 3.2. Red Deer

Contrary to DRB genotyping in roe deer, in red deer, the alleles detected by different methods varied to some extent in most individuals. Out of 30 colonies collected per individual, an average of 17.7 was successfully sequenced and assigned to individual alleles (Table 2). Discarded sequences were either chimeras or poor quality sequences. Cloning failed to detect alleles in six samples (ten alleles in total, seven of them unique) in comparison to combined genotypes. Alleles that were found at high frequencies using NGS methods were generally detected in cloning as well. However, some alleles were still not detected despite being found in relatively high frequencies in NGS analyses (e.g., allele Ceel-DRB*HR17 was found at a frequency of 27% in web Illumina analysis and allele Ceel-DRB*HR26 was found at a frequency of 14.8% in Illumina web and 22.5% in Ion Torrent web analysis) (Table 2). 

In total, molecular cloning was able to detect 32 alleles across all individuals, which equals 76.2% of the total number of combined genotypes detected (Table 3). The number of alleles per individual genotype found by cloning/Sanger sequencing ranged from 2 to 5.

Our first high-throughput analyses of red deer data, which included the AmpliSAS web tool (which uses a subset of 5000 reads), failed to obtain a perfect match among three genotyping methods, so we further analysed NGS data on red deer using the AmpliSAS script installed locally to examine the whole dataset. All 10 samples reached a coverage of markedly over 5000 reads in both Ion Torrent and Illumina amplicon sequencing (Table 4). The two NGS platforms generated 3,891,407 reads in total, with 1,298,414 belonging to Illumina sequencing and 3,891,407 to Ion Torrent. A total number of 2,766,525 reads was kept after AmpliCLEAN length and quality filtering. The average proportion of reads assigned to alleles was higher in Ion Torrent data (79.2% in local and 80.8% in web analysis) and lower in Illumina data (72.3% in local and 72.4% in web analysis), but similar between AmpliSAS web and local analyses of the particular platform (Table 4).

Illumina sequencing resulted in 1,227,184 reads after size and quality filtering, while the average number of reads per sample equalled 123,089. The proportion of reads assigned to alleles was very similar between local and web AmpliSAS analyses for each sample, ranging from 61.8% to 83.6% obtained in local and from 62.1% to 83.6% obtained in web analyses (Table 4), as well as allele frequencies within each sample (Table 2). A complete genotype match was observed between the web and local AmpliSAS analysis of Illumina data (Table 3). An overall number of 42 alleles was detected across all individuals, which is the maximum number of detected genotypes in all methods and therefore corresponds to 100% of the combined genotypes detected. In other words, no allelic dropouts were detected in comparison to combined genotypes (Table 3). The number of alleles per individual genotype ranged from 2 to 6. All alleles were found at frequencies of >3%, apart from allele CeelHap103 in the sample J16B.

Ion Torrent sequencing generated 1,539,341 reads after size and quality filtering. The average number of reads per sample was 145,433. Although the average proportion of reads assigned to alleles was very similar between local and web AmpliSAS analyses (79.2 and 80.8%, respectively), it differed quite substantially in some samples (Table 4). For example, in sample 28, 67.1% of reads were assigned to alleles in web analyses while as much as 75.5% were assigned in local analyses, while sample J2GK showed the opposite pattern, with a lower proportion of reads assigned to alleles in local analysis (81.3%) and a higher proportion (88.0%) assigned in the web analysis. In addition, in samples J16B, J29B, and J30B, allele frequencies differed substantially between web and local analyses (Table 2). Most importantly, in some samples, web analysis entirely missed particular alleles found at various frequencies by local analysis, resulting in a different number of alleles detected across individuals between the two AmpliSAS analyses. On the whole, the local analysis scored 81.0% of the combined genotypes (34 alleles), while web analysis scored only 69.0% of the combined genotypes (29 alleles) (Table 3). The number of alleles per individual genotype ranged from two to five in local analysis, and in web analysis, from two to four. Allelic dropout was observed in eight samples after Ion Torrent web analysis with 13 undetected alleles. However, local analysis succeeded in obtaining more complete allelic profiles, as eight alleles remained undetected in six samples (Table 2).

The average allele frequency (average frequency of reads corresponding to each allele across the whole sample set) obtained in AmpliSAS local analyses ranged from 2.7% to 67.8% for Illumina and from 1.0% to 84.8% for Ion Torrent (Supplementary file: Table S2). Allele Ceel-DRB*HR06 had the highest average allele frequency after both Illumina and Ion Torrent analysis. However, average allele frequency obtained after Illumina and Ion Torrent sequencing varied substantially for some alleles. For instance, allele Ceel-DRB*HR02 had an average frequency of 48.5% in Ion Torrent analysis, but only 20.1% in Illumina. The most extreme case is the allele with the second-highest average allele frequency in Illumina analysis (27.8%, allele Ceel-DRB*HR24) that was not detected with Ion Torrent.

Finally, the analysis of combined genotypes resulted in the classification of 19 unique alleles, including five newly discovered alleles that were deposited in the GenBank (accession numbers ON204043-ON204047). All of the alleles had an identical length of 249 bp and could be translated into unique amino acid sequences (Supplementary file: Figure S3).

## 4. Discussion

In our work, we aimed to compare the utility of different approaches in genotyping the MHC-DRB locus in species with a simple MHC system, such as the European roe deer, which has a single copy of the gene, and in red deer, which is a species with multiple DRB loci. Our results emphasise the need for a species-specific methodological approach, even when genotyping closely related mammalian species whose MHC genes might be quite complex but not as complex as in some other mammalian species (e.g., MHC class I in Iberian lynx [8] or other vertebrate taxa, such as birds [30,31,32,33]). Using multiple parallel methods in preliminary allele assessment offers the possibility to test which of the methods is the most appropriate for future genotyping projects, and it can also serve for validation of allele findings, as detecting an allele in a single individual with multiple approaches further supports its credibility. In this research, we evaluated the performance of three different genotyping methods (cloning/Sanger sequencing, Illumina MySeq, and Ion Torrent S5 System) for MHC-DRB genotyping and contrasted them between two species with a different number of DRB copies—roe and red deer. In an effort to avoid complex bioinformatic analyses, we inspected the utility of an easily operated web version of the AmpliSAS pipeline, which only considers the first 5000 sequence reads. We demonstrated that 5000 reads are sufficient for the assessment of DRB alleles in species with a single locus (roe deer), regardless of the NGS platform used. A complete genotype match was observed between roe deer results from both Illumina and Ion Torrent platforms, and genotypes were further confirmed by cloning/Sanger sequencing (Table 1). However, in red deer, a species with as many as four DRB loci [13,16], 5000 reads (as used in web AmpliSAS analysis) proved to be insufficient for Ion Torrent analyses (Table 2 and Table 3). An increase in coverage gained with the local AmpliSAS analysis (as it was performed on all available reads with a maximum of 200,000 reads) improved the degree of concordance between the results of Ion Torrent sequencing and combined genotypes, but not to a complete genotype match.

With Illumina sequencing, a maximum number of alleles was detected across all samples (42), 39 of which were confirmed with either molecular cloning or Ion Torrent sequencing (Table 2). The remaining three allele findings represented two unique alleles that had the lowest average allele frequency found by Illumina (Ceni-DRB*24 and CeelHap103, Table 2 and Table S2), which presumably indicates their lower amplification efficacy. Although allele Ceni-DRB*24 was detected by only one genotyping approach, i.e., Illumina sequencing, we found it quite reliable as it was present in two individuals and still not in extremely low frequencies (they were above 4%) (Table 2). In addition, it was not a newly found allele as it was detected previously in Scottish red deer populations [17]. Apart from Ceni-DRB*24, allele CeelHap103 was found at a low frequency in Illumina analyses (2.0–3.2%) and was not detected with molecular cloning. Nonetheless, it was found by Ion Torrent sequencing as well (in sample J29B) (Table 2), contributing to its reliability. To sum up, although both traditional and high-throughput sequencing methods relying on PCR are prone to the formation of chimeric sequences and the unbalanced amplification of alleles [3,6], we ruled out the possibility that some of the detected alleles were artificial sequences, as they were either confirmed by more than one genotyping method (cloning/Sanger sequencing, Illumina, and/or Ion Torrent) or found in multiple individuals (Table 2). Generally, the comparison of methods and individual allelic profiles in this research helped us validate detected alleles.

The second most accurate genotyping approach was the Ion Torrent sequencing analysed with the AmpliSAS pipeline, which takes into account all available amplicon reads, followed by molecular cloning. Results obtained after AmpliSAS analysis of the first 5000 Ion Torrent sequencing data (web version) were the least accurate, with 69% combined genotypes detected (Table 3). Molecular cloning combined with Sanger sequencing represents a traditional method, often still considered to be “the gold standard” for allele assessment. Due to amplification bias, it is often challenging to estimate the required number of clones. Our cloning results suggest that even 30 recombinant clones per individual might not be sufficient for the successful identification of complete genotypes in a species with multiple DRB genes, especially if a substantial number of recombinant colonies produce unsatisfactory sequences. By sequencing 48 recombinant colonies per individual, Pérez-Espona et al. [17] managed to show congruence with genotypes assigned using NGS. However, sequencing a larger number of positive colonies inevitably increases the manual labour as well as the cost of the analysis. On the other hand, for single-locus species, 15 recombinant clones per individual proved sufficient to detect both alleles in heterozygous animals, which is in line with previous research (e.g., [13,34]). Non-consistency in results across NGS platforms was previously acknowledged [9,35,36,37] and could be attributed to various factors such as differences in sequencing chemistry, library preparation protocols [9], or it could even be run-specific, as implied by Grogan et al. [18].

## 5. Conclusions

As high-throughput sequencing technologies are increasingly utilised in amplicon sequencing projects such as MHC genotyping, the present study contributes to adopting an optimal strategy for reliable detection of allelic profiles, which does not include complex bioinformatic analyses. In conclusion, we found both high-throughput methods to work similarly well in single-locus species, but in this research, Illumina showed somewhat higher performance in multi-locus species, as more complete genotypes were obtained by this approach.

## Figures and Tables

**Table 1:** Number of detected sequences with molecular cloning (Clon) and frequencies of reads corresponding to each allele (%) obtained using AmpliSAS web version after Illumina (ILL) and Ion Torrent (IT) sequencing of 14 European roe deer at the MHC-DRB locus.

Sample ID	1SL			1SN			2SL			3SL			4SC			12SC			13SC		
ALLELES	Clon	ILL	IT	Clon	ILL	IT	Clon	ILL	IT	Clon	ILL	IT	Clon	ILL	IT	Clon	ILL	IT	Clon	ILL	IT
Caca-DRB*0102	2	43.44	34.48	2	40.42	41.06	3	38.78	34.38	1	39.62	34.34	2	40.06	41.34						
Caca-DRB*0301																4	47.92	20.16	1	43.40	30.14
Caca-DRB*0302				1	41.36	42.78							2	44.16	44.50						
Caca-DRB*0304																4	43.76	68.92			
Caca-DRB*0401																			3	39.24	55.58
Caca-DRB*0402							4	41.78	48.62												
Caca-DRB*0403	3	41.32	52.08							4	31.60	48.34									
Caca-DRB*0405																					
Sample ID	14SC			K7			K10			L2			L5			L19			L20		
ALLELES	Clon	ILL	IT	Clon	ILL	IT	Clon	ILL	IT	Clon	ILL	IT	Clon	ILL	IT	Clon	ILL	IT	Clon	ILL	IT
Caca-DRB*0102	4	46.02	51.52				1	42.82	37.02												
Caca-DRB*0301	2	48.26	35.22										3	42.64	20.62	2	42.56	23.04	4	47.32	27.12
Caca-DRB*0302							1	42.08	48.82										3	50.70	60.96
Caca-DRB*0304				5	41.30	55.46															
Caca-DRB*0401													3	41.70	64.54	5	39.14	61.26			
Caca-DRB*0402				3	46.24	28.60				2	54.06	41.68									
Caca-DRB*0403																					
Caca-DRB*0405										3	43.08	48.04									

**Table 2:** Number of detected sequences with molecular cloning (Clon) and frequencies of reads corresponding to each allele (%) obtained using AmpliSAS web and local versions after Illumina (ILL) and Ion Torrent (IT) sequencing of 10 red deer at MHC-DRB loci.

Sample ID	J2GK					J9GK					J16B					J20B				
	Clon	ILL		IT		Clon	ILL		IT		Clon	ILL		IT		Clon	ILL		IT	
**ALLELES**		**web**	**local**	**web**	**local**		**web**	**local**	**web**	**local**		**web**	**local**	**web**	**local**		**web**	**local**	**web**	**local**
Ceel-DRB*HR02	10	18.58	18.43	63.54	58.67											3	12.52	11.65	37.04	31.76
Ceel-DRB*HR04											6	24.30	24.29	80.22	48.47	5	15.28	15.25	36.40	30.38
Ceel-DRB*HR06						22	67.00	67.54	86.44	84.77										
Ceel-DRB*HR09																				
Ceel-DRB*HR10	4	23.20	24.06	24.48	22.63															
Ceel-DRB*HR11																				
Ceel-DRB*HR12											4	29.00	29.49	5.24	31.48	4	19.86	20.05	13.66	21.73
Ceel-DRB*HR16																				
Ceel-DRB*HR17																1	7.86	7.59	2.22	2.86
Ceel-DRB*HR21																				
Ceel-DRB*HR24	6	27.48	27.77																	
Ceel-DRB*HR25											3	11.98	11.71			2	66.0	7.30		
Ceel-DRB*HR26																				
Ceni-DRB*12																				
Ceni-DRB*14																				
Ceel-DRb*HR27																				
Ceel-DRB*HR28						1	16.56	16.02		1.95										
Ceni-DRB*24												4.22	4.70							
CeelHap103												2.00	2.24							
**Sample ID**	**J24B**					**J25B**					**J29B**					**J30B**				
	**Clon**	**ILL**		**IT**		**Clon**	**ILL**		**IT**		**Clon**	**ILL**		**IT**		**Clon**	**ILL**		**IT**	
**ALLELES**		**web**	**local**	**web**	**local**		**web**	**local**	**web**	**local**		**web**	**local**	**web**	**local**		**web**	**local**	**web**	**local**
Ceel-DRB*HR02						19	30.50	30.13	65.88	55.20										
Ceel-DRB*HR04	5	13.36	12.78	41.10	22.65											2	26.32	25.82	85.38	45.74
Ceel-DRB*HR06																				
Ceel-DRB*HR09																7	15.18	15.21	1.30	14.50
Ceel-DRB*HR10																				
Ceel-DRB*HR11						2	19.40	20.12	12.26	16.25										
Ceel-DRB*HR12	8	18.14	17.83	20.74	23.41											5	15.30	15.14		13.48
Ceel-DRB*HR16		10.74	11.09	11.00	11.44						11	20.28	20.68	40.96	27.67					
Ceel-DRB*HR17							26.96	26.29	8.18	10.20										
Ceel-DRB*HR21		7.96	8.76		6.90						4	19.94	18.79	6.74	19.51					
Ceel-DRB*HR24																				
Ceel-DRB*HR25	1	5.46	5.84													2	9.26	9.52		
Ceel-DRB*HR26		8.32	8.41	3.28	6.57						3	21.46	21.64	18.68	27.67					
Ceni-DRB*12																	8.34	8.37		6.04
Ceni-DRB*14																				
Ceel-DRb*HR27																				
Ceel-DRB*HR28																				
Ceni-DRB*24												4.96	4.66							
CeelHap103												3.10	3.16	5.62	1.03					
**Sample ID**	**24**					**28**														
	**Clon**	**ILL**		**IT**		**Clon**	**ILL**		**IT**											
**ALLELES**		**web**	**local**	**web**	**local**		**web**	**local**	**web**	**local**										
Ceel-DRB*HR02																				
Ceel-DRB*HR04																				
Ceel-DRB*HR06																				
Ceel-DRB*HR09																				
Ceel-DRB*HR10																				
Ceel-DRB*HR11	2	15.22	15.15	33.38	18.11															
Ceel-DRB*HR12																				
Ceel-DRB*HR16						3	11.74	11.27	31.56	13.47										
Ceel-DRB*HR17																				
Ceel-DRB*HR21	8	35.06	33.96	9.66	30.81	4	11.96	12.82	8.80	14.36										
Ceel-DRB*HR24																				
Ceel-DRB*HR25																				
Ceel-DRB*HR26	10	33.06	33.13	27.28	25.69		14.80	14.68	22.46	16.25										
Ceni-DRB*12																				
Ceni-DRB*14						7	17.08	16.54	4.28	19.67										
Ceel-DRb*HR27						3	13.96	13.42		11.78										
Ceel-DRB*HR28																				
Ceni-DRB*24																				
CeelHap103																				

**Table 3:** MHC-DRB combined genotypes (number of combined alleles), number of alleles detected in individuals, and proportion of the combined genotype detected by different methods (P). ILL web—Illumina web, ILL local—Illumina local, IT web—Ion Torrent web, IT local—Ion Torrent local analysis, cloning—cloning/Sanger sequencing method.

Sample ID	J2GK	J9GK	J16B	J20B	J24B	J25B	J29B	J30B	24	28	Total	
Combined genotypes	3	2	5	5	6	3	5	5	3	5	42	P (%)
ILL web	3	2	5	5	6	3	5	5	3	5	42	100.0
ILL local	3	2	5	5	6	3	5	5	3	5	42	100.0
IT web	2	1	2	4	4	3	4	2	3	4	29	69.0
IT local	2	2	2	4	5	3	4	4	3	5	34	81.0
Cloning	3	2	3	5	3	2	3	4	3	4	32	76.2

**Table 4:** The number of reads per sample generated with Illumina and Ion Torrent sequencing of MHC-DRB in red deer after AmpliCLEAN filtering and the proportion of reads assigned to alleles. After size and quality filtering, Illumina generated 1,227,184 and Ion Torrent generated 1,539,341 reads in total.

	Illumina			Ion Torrent		
Sample ID	Total No. of Reads	Web	Local		Web	Local
		Proportion of Reads Assigned to Alleles (%)	Proportion of Reads Assigned to Alleles (%)	Total No. of Reads	Proportion of Reads Assigned to Alleles (%)	Proportion of Reads Assigned to Alleles (%)
J2GK	114,148	69.3	70.3	135,524	88.0	81.3
J9GK	112,369	83.6	83.6	156,880	86.4	86.7
J16B	140,322	71.5	72.4	163,377	85.5	80.4
J20B	96,327	62.1	61.8	76,560	89.3	86.7
J24B	159,466	64.0	64.7	200,000	76.1	71.0
J25B	112,820	76.9	76.6	200,000	86.3	81.7
J29B	88,785	69.7	68.9	137,417	72.0	73.9
J30B	155,793	74.4	74.1	180,089	86.7	79.8
24	111,666	83.3	82.2	85,775	70.3	74.6
28	139,196	69.5	68.7	118,711	67.1	75.5
average	123,089	72.4	72.3	145,433	80.8	79.2

## Data Availability

Newly discovered MHC alleles have been imported to GenBank (accession numbers: ON204042-ON204047).

## Supplementary Materials

The following supporting information can be downloaded at: https://www.mdpi.com/article/10.3390/ani12182452/s1, Table S1: The number of reads per sample generated with Illumina and Ion Torrent sequencing of MHC-DRB in European roe deer after AmpliCLEAN filtering, and the proportion of reads as-signed to alleles. After size and quality filtering, Illumina generated 1,202,786, and Ion Torrent 876,347 reads in total; Table S2: Average allele frequency (average frequency of reads corresponding to each allele across the whole sample set) obtained for Illumina and Ion Torrent AmpliSAS local analysis of red deer samples. The frequencies are given in descending order in the Illumina column and newly found alleles are underlined; Figure S1: MHC-DRB allele frequency ratios detected in European roe deer by utilisation of Illumina (left bar) and Ion Torrent (right bar) sequencing followed by AmpliSAS web analysis (a subset of 5000 reads); Figure S2: Alignment of the MHC-DRB alleles detected in 14 European roe deer, identities are plotted to first sequence with a dot; Figure S3: Alignment of the MHC-DRB alleles detected in 10 red deer, identities are plotted to first sequence with a dot.
