# Supplementary material for: Performance Comparison of Different Approaches in Genotyping MHC-DRB: The Contrast between Single-Locus and Multi-Locus Species
Source: Animals (Basel). 2022 Sep 16;12(18):2452. doi: 10.3390/ani12182452 (PMC9495155; doi:10.3390/ani12182452)
Supplement: Supplementary file 1 [file animals-12-02452-s001.zip › animals-1886779-supplementary.pdf]

Table S1. The number of reads per sample generated with Illumina and Ion Torrent sequencing of MHC-DRB in European roe deer after AmpliCLEAN filtering, and the proportion of reads assigned to alleles. After size and quality filtering, Illumina generated 1,202,786, and Ion Torrent 876,347 reads in total.

	ILLUMINA		ION TORRENT	
Sample	Total no. of reads	Proportion of reads assigned to alleles (%)	Total no. of reads	Proportion of reads assigned to alleles (%)
1SL	64,741	84.8	46,284	86.6
1SN	67,351	81.8	43,199	83.8
2SL	105,622	80.6	84,030	83.0
3SL	83,552	71.2	59,798	82.7
4SC	59,407	84.2	59,520	85.8
12SC	73,516	91.7	112,812	89.1
13SC	94,244	82.6	63,530	85.7
14SC	109,486	94.3	37,696	86.7
K7	72,908	87.5	115,259	84.1
K10	69,648	84.9	41,700	85.8
L2	112,464	97.1	64820	89.7
L5	122,904	84.3	58,535	85.2
L19	68,869	81.7	67,586	84.3
L20	98,074	98.0	21,578	88.1
AVG	85,913	86.1	62,596	85.8

Table S2. Average allele frequency (average frequency of reads corresponding to each allele across the whole sample set) obtained for Illumina and Ion Torrent AmpliSAS local analysis of red deer samples. The frequencies are given in descending order in the Illumina column and newly found alleles are underlined.

Illumina local (%)	Alleles	Ion Torrent local (%)
67.5	Ceel-DRB*HR06	84.8
27.8	<u>Ceel-DRB*HR24</u>	-
24.1	Ceel-DRB*HR10	22.6
20.6	Ceel-DRB*HR12	22.5
20.1	Ceel-DRB*HR02	48.5
19.5	Ceel-DRB*HR04	36.8
19.5	<u>Ceel-DRB*HR26</u>	19.0
18.6	Ceel-DRB*HR21	17.9
17.6	Ceel-DRB*HR11	17.2
16.9	Ceel-DRB*HR17	6.5
16.5	Ceni-DRB*14	19.7
16.0	<u>Ceel-DRB*HR28</u>	1.9
15.2	Ceel-DRB*HR09	14.5
14.3	Ceel-DRB*HR16	17.5
13.4	<u>Ceel-DRb*HR27</u>	11.8
8.6	<u>Ceel-DRB*HR25</u>	-
8.4	Ceni-DRB*12	6.0
4.7	Ceni-DRB*24	-
2.7	CeelHap103	1.0

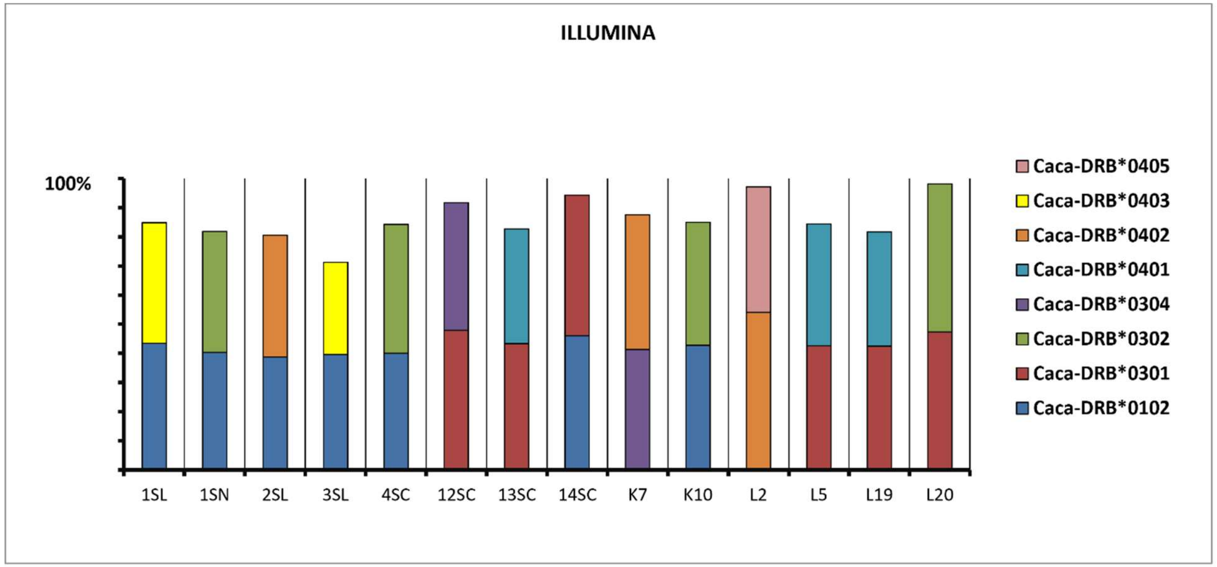

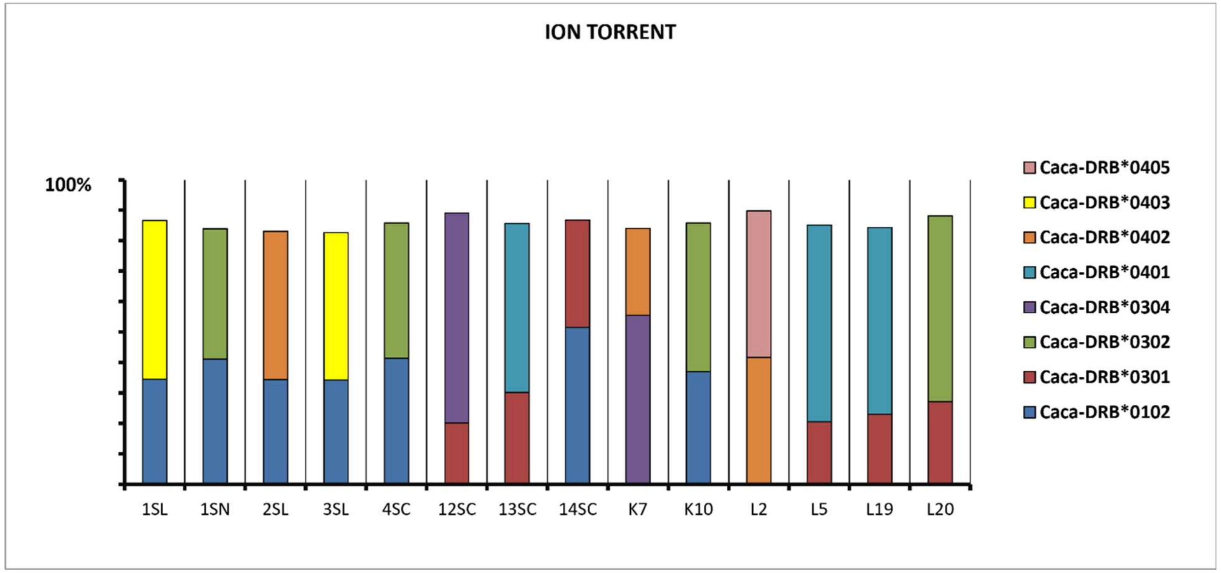

Figure S1 MHC-DRB allele frequency ratios detected in European roe deer by utilisation of Illumina (left bar) and Ion Torrent (right bar) sequencing followed by AmpliSAS web analysis (a subset of 5,000 reads)

```
                ....|....| ....|....| ....|....| ....|....| ....|....| ....|....| ....|....| ....|....| ....|....| ....|....|
                        10         20         30         40         50         60         70         80         90        100
Caca-DRB*0102   ATGTATACTA CGGGCGAGTG TCATTTCTCC AACGGGACGG AGCGGGTGCG GTTCCTGGAC AGATACTTCT ATAACGGAGA AGAGTTCGTG CGCTTCGACA
Caca-DRB*0301   .......... ..A....... .......... .......... .......... .......... .......... .......... .....A.... ..........
Caca-DRB*0302   .......... A.AAA..... .T........ .......... .......... .......... .......... .......... .....A.... ..........
Caca-DRB*0304   .......... ..A....... .......... .......... .......... .......... .......... .......... .....A.... A.........
Caca-DRB*0401   .......... A.AAA..... .T........ ..T....... .......... .......... .......... .......... .......... ..........
Caca-DRB*0402   .......... A.AAA..... .T........ ..T....... .......... .......... .......... .......... .....A.... ..........
Caca-DRB*0403   .......... A.AAA..... .T........ ..T....... .......... .......... .......... .......... .......... ..........
Caca-DRB*0405   .......... A.AAA..... .T........ ..T....... .......... .......... .......... .......... .......... ..........

                ....|....| ....|....| ....|....| ....|....| ....|....| ....|....| ....|....| ....|....| ....|....| ....|....|
                       110        120        130        140        150        160        170        180        190        200
Caca-DRB*0102   GCGACTGGGG CGAGTACCGG GCGGTGACCG AGCTGGGGCG GCCGGTGGCC GAGGGCTGGA ACAGCCAGAA GGAGTTCCTG GAGCAGAGGC GGGCCGAGGT
Caca-DRB*0301   .......... .......... .......... .......... ...C.AC... A..TA..... ........-- -...A..... ....G.G... ......C...
Caca-DRB*0302   .......... .......... .......... .......... ...C.AC... A..TA..... ........G. .A---..... ....G.G... ......C...
Caca-DRB*0304   .......... .......... .......... .......... ....TCC... A..TA..... .......... ....A..... .......... ......C...
Caca-DRB*0401   .......... .......... .......... .......... .......... .......... .......... .......... .......... ..........
Caca-DRB*0402   .......... .......... .......... .......... ...C.AC... A..TA..... ........G. .A---..... ....G.G... ......C...
Caca-DRB*0403   .......... .......... .......... .......... .....AC... A..TA..... .......... .......... ....G.G... ......C...
Caca-DRB*0405   .......... .......... .......... .......... .....AC... A..TA..... .......... .......... ....G.G... ......C...

                ....|....| ....|....| ....|....| ....|....| ....|....
                       210        220        230        240
Caca-DRB*0102   GGACACGTAC TGCAGACACA ACTACGGGGT CGGTGAGAGT TTCACTGTG
Caca-DRB*0301   .......... .......... .......... TAT....... .........
Caca-DRB*0302   .......... .......... .......... TAT....... .........
Caca-DRB*0304   .......... .......... .......... TAT....... .........
Caca-DRB*0401   .......... .......... .......... .......... .........
Caca-DRB*0402   .......... .......... .......... TAT....... .........
Caca-DRB*0403   .......... .......... .......... .......... .........
Caca-DRB*0405   .......... .......... .......... TAT....... .........
```

Figure S2 Alignment of the MHC-DRB alleles detected in 14 European roe deer, identities are plotted to first sequence with a dot.

```
                ....|....| ....|....| ....|....| ....|....| ....|....| ....|....| ....|....| ....|....| ....|....| ....|....|
                        10         20         30         40         50         60         70         80         90        100
Ceel-DRB*HR02   GAGTATCATA AGAGCGAGTG TCATTTCTCC AACGGGACGC AGCGGGTGGG GTACCTGGAG AGATACATCT ATAACCGGGA AGAGTACGTG CGCTTCGACA
Ceel-DRB*HR04   ......GC.. .......... .......... .......... ........C. ..T......C ......T... .......... .......... ..........
Ceel-DRB*HR06   ......T.C. .......... C.......T. .........G ........C. ..T....C.C ......T.T. C...TG.A.. ....AC.... ..........
Ceel-DRB*HR09   ...C...T.. ..GC...... ........T. .........G .....A..CA ..T.....C. ......T... .....G.A.. ........C. ..........
Ceel-DRB*HR10   CT....AC.. C......... .......... .......... ........CA ..T......C ......T... .....G.A.. .....T.... ..........
Ceel-DRB*HR11   CT....AC.. C.G....... .......... .......... .......... ..T......C ......T... .....G.A.. .......... ..........
Ceel-DRB*HR12   ...C...T.. ..GC...... ........T. .........G .....A..CA ..T.....C. ......T... .....G.A.. ........C. ..........
Ceel-DRB*HR16   CT....GC.. .......... .......... .......... .......... ..T......C ......T... .....G.A.. .....T.... ..........
Ceel-DRB*HR17   CT.C..AT.. ..GC...A.. .......... .......... .......... .......... ...C...... .....G.A.. .....T.C.. ..........
Ceel-DRB*HR21   CT....AC.. C......... .......... .......... .......... ..T......C ......T... .....G.A.. .......... ..........
Ceel-DRB*HR24   ......TTC. .......... .......... .........G ........C. .CT......C ......G... .......... .......... ....A.....
Ceel-DRB*HR25   AT....AC.. ...AA..... .......... .......... .......... .CT......C ......T... .....G.A.. .....T.... ..........
Ceel-DRB*HR26   ...C...... ..T....... .......... .........G ........CA ..T....C.. .......... .......... .......... ..........
Ceel-DRB*HR27   CT....AC.. C......... .......... .......... .......... ..T......C ......T... .....G.A.. .......... ..........
Ceel-DRB*HR28   CT....AC.. C......... .......... .......... .......... ..T......C ......T... .....G.A.. .......... ..........
Ceni-DRB*24     ......GC.. ....A..... .......... .......... .......... ..T......C ......T... .....G.A.. .......... ..........
CeelHap103      ......T.C. ..C....... ........T. .......... .......... ..T......C ......T.T. C...TG.A.. ....AC.... ..........
Ceni-DRB*12     AT....AC.. ...AA..... .......... .......... .......... .CT......C ......T... .....G.A.. .....T.... ..........
Ceni-DRB*14     ...C.G.T.. ..G....... .......... .......... ........C. .CT....C.. .......... .......... ........C. ....A.....

                ....|....| ....|....| ....|....| ....|....| ....|....| ....|....| ....|....| ....|....| ....|....| ....|....|
                       110        120        130        140        150        160        170        180        190        200
Ceel-DRB*HR02   GCGACTGGGG CGAGTACCGG GCGGTGACCG AGCTGGGGCG GCCGTCTGCC AAGTACATGA ATAGCCAGAA GGAGCTCCTG GAGCGGAAGC GGGCCAATGT
Ceel-DRB*HR04   .......... .....T.... .......... .......... ......C... ......TG.. .C........ ...TT..A.. ....A..... .....G.G..
Ceel-DRB*HR06   .....GT... .....T.... .......... .......... ....GAC... G..CT.TG.. .C..T..... ...CACT... ...GACG.A. .....GCA..
Ceel-DRB*HR09   .....GT... .....T.... .......... .......... ....GAC... ......TG.. .C........ ....A..... ....A.C.CG ....AG.G..
Ceel-DRB*HR10   .......... .......... ........A. .......... ....G.G... G..GG.TG.. .C..A..... ....T..... ....A.GC.. .....GCG..
Ceel-DRB*HR11   .......... ......T... .......... .......... ....GAC... G..CT.TG.. .C........ ....T..... ....A..G.. .....G.G..
Ceel-DRB*HR12   .....GT... .....T.... .......... .......... ....GAC... ......TG.. .C........ ....A..... ....A.C.C. ....AG.G..
Ceel-DRB*HR16   .......... .......... ........A. .......... ....GTG... G.....C... .C........ ....TA.A.. ....A..C.. .....G.G..
Ceel-DRB*HR17   .....GT... .......... .......... .......... T...GAG... ....C.TG.. .C........ .......... ...GA..G.. .....GCG..
Ceel-DRB*HR21   .......... .......... ........A. .......... ......C... ......TG.. .C........ ....TA.A.. ....A..C.. .....G.G..
Ceel-DRB*HR24   .....GT... ........A. ........A. .......... ......C... ......TAT. .C........ .......... ...GAC..C. .....GCG..
Ceel-DRB*HR25   .......... .....T.... .......... .......... ....G.G... G..GG.TG.. .C........ .......... ....A..G.. .....GCG..
Ceel-DRB*HR26   .......... .......... ........A. .......... ......C... ......TAT. .C........ .......... ....A..... .....GCG..
Ceel-DRB*HR27   .......... .......... ........A. .......... ......C... ......TG.. .C........ ....TA.A.. ....A..C.. .....G.G..
Ceel-DRB*HR28   .......... ......T... ........A. .......... ....GAC... ......TG.. .C........ ....TA.A.. ......GC.. .....GCG..
Ceni-DRB*24     .......... ......T... ........A. .......... ......C... ......TG.. .C........ ....TA.A.. ......GC.. .....GCG..
CeelHap103      .......... .......... ........A. .......... ...CGA.... G..CT.TG.. .C........ ....A..... ......GC.. .....GCG..
Ceni-DRB*12     .......... .....T.... .......... .......... ....GAC... G..GG.TG.. .C..A..... .......... ....A..G.. .....GCG..
Ceni-DRB*14     .......... .....T.... ........A. .......... ....GAC... G.....TG.. .C........ ....TA.A.. ...GA.TT.. .....GCG..

                ....|....| ....|....| ....|....| ....|....| ....|....
                       210        220        230        240
Ceel-DRB*HR02   GGACACGTAC TGCAGATACA ACTACGGGGT TATTGAGAGT TTCACTGTG
Ceel-DRB*HR04   .......GTG ......C... .......... .......... .........
Ceel-DRB*HR06   ........T. ......C... .....C.... CGG....... .........
Ceel-DRB*HR09   .....G.... ......C... .......... CGG....... .........
Ceel-DRB*HR10   .......... ......C... .......C.G CG........ .........
Ceel-DRB*HR11   .......... ......C... .......... CGG....... .........
Ceel-DRB*HR12   .....G.... ......C... .......... CGG....... .........
Ceel-DRB*HR16   .......... ......C... .......C.G CG........ .........
Ceel-DRB*HR17   .A........ .......... .......... CGG....... .........
Ceel-DRB*HR21   .....G.... ......C... .......... .C....C... ...G.....
Ceel-DRB*HR24   .......... ......C... .......... CGG....... .........
Ceel-DRB*HR25   .......... ......C... .......... .......... .........
Ceel-DRB*HR26   .....G.... ......C... .......... CG........ .........
Ceel-DRB*HR27   .....G.... ......C... .......... .......... ...G.....
Ceel-DRB*HR28   .......... ......C... .......C.G CG........ .........
Ceni-DRB*24     .......... ......C... .......... CGG....... .........
CeelHap103      .......... ......C... .......... .T........ .........
Ceni-DRB*12     .......... ......C... .......... .......... .........
Ceni-DRB*14     .......... ......C... .......... CGG....... .........
```

Figure S3. Alignment of the MHC-DRB alleles detected in 10 red deer, identities are plotted to first sequence with a dot.
